# European research trends in nuclear medicine

**DOI:** 10.1007/s12149-018-1303-7

**Published:** 2018-09-21

**Authors:** Masayuki Inubushi, Mitsuaki Tatsumi, Yuka Yamamoto, Katsuhiko Kato, Tetsuya Tsujikawa, Ryuichi Nishii

**Affiliations:** 1 Division of Nuclear Medicine, Department of Radiology, Kawasaki Medical School, 577 Matsushima, Kurashiki, Okayama 701-0192 Japan; 2 Department of Radiology, Osaka University Hospital, 2-2 Yamadaoka, Suita, Osaka 565-0871 Japan; 3 Department of Radiology, Faculty of Medicine, Kagawa University, 1750-1 Ikenobe, Miki-cho, Kita-gun, Kagawa, 761-0793 Japan; 4 Department of Radiological and Medical Laboratory Sciences, Nagoya University Graduate School of Medicine, 1-20, Daikominami 1-chome, Higashi-ku, Nagoya, Aichi 461-8673 Japan; 5 Biomedical Imaging Research Center, University of Fukui, 23-3 Matsuoka-Shimoaizuki, Eiheiji-cho, Fukui, 910-1193 Japan; 6 Department of Molecular Imaging and Theranostics, National Institute of Radiological Sciences (NIRS), QST, 4-9-1 Anagawa, Inage-ku, Chiba, 263-8555 Japan

**Keywords:** Treatment response, Hypoxia PET, Sentinel lymph node biopsy, Amyloid PET, Rodents

## Abstract

Written by associate editors of the Annals of Nuclear Medicine, this invited review article is intended to offer our readers a condensed global view on the high-quality research work that has been published in Europe last year. We have divided this article into five sections. The first three sections from the oncology category include “[^18^F]fluorodeoxyglucose (FDG) positron-emission tomography (PET) for therapy monitoring in malignant lymphoma”, “[^18^F]fluoromisonidazole (FMISO) PET for hypoxia”, and “lymphoscintigraphy update”. It is followed by a section on “amyloid PET for Alzheimer’s disease” using [^11^C]Pittsburgh Compound B (PiB) and [^18^F]florbetapir from the neurology category. The final section reviews three original articles in the field of “basic and translational molecular imaging” regardless of the category, which investigated new PET tracers such as L-4-borono-2-[^18^F]fluoro-phenylalanine (FBPA), *O*-(2-[^18^F]fluoroethyl)-L-tyrosine (FET) and ^64^Cu-NOTA-pertuzumab in small animals. We hope that this review article will arouse greater interest in our readers in recent European research trends in the field of nuclear medicine.

## Introduction

This review article is intended to offer our readers a condensed global view on the high-quality research work that has been published in Europe last year [1]. We have divided this article into five sections. The first three sections from the oncology category include “[^18^F]fluorodeoxyglucose (FDG) positron-emission tomography (PET) for therapy monitoring in malignant lymphoma”, “[^18^F]fluoromisonidazole (FMISO) PET for hypoxia”, and “lymphoscintigraphy update”. It is followed by a section on “amyloid PET for Alzheimer’s disease” from the neurology category. The final section reviews three original articles in the field of “basic and translational molecular imaging” regardless of the category, which investigated new PET tracers in small animals.

## Oncology—FDG PET for therapy monitoring in malignant lymphoma

FDG PET has been used to evaluate treatment response in various malignancies. Malignant lymphoma, among others, is one of the major targets for this indication. Response assessment with FDG PET has officially been accepted in lymphoma since Cheson et al. [2] announced “Revised response criteria for malignant lymphoma” in 2007. Barrington and Kluge [3] reviewed published data regarding therapy monitoring in Hodgkin and non-Hodgkin lymphomas (HL and NHL, respectively) with FDG PET. This review article included 6 sections as follows: FDG PET for monitoring therapy and the development of the Deauville criteria, Application and use of the Deauville criteria, Quantification in response monitoring, Quantitative methods in oncology, Response-adapted treatment using FDG PET, and Role in radiation therapy (RT) planning. They introduced and emphasized the important role of the Deauville five-point scale, a visual assessment method adopted in the Lugano classification, in response assessment of HL and NHL including T-cell lymphomas [4]. They also mentioned several quantitative PET parameters to monitor response and response-adopted treatment using the interim PET findings.

Interim FDG PET performed after one to four cycles of chemotherapy is increasingly being considered as a promising approach to stratify lymphoma patients who are expected to show a treatment response. However, the value of interim PET to predict outcome is still controversial due to its high false-positive rate. Lazarovici et al. [5] reported the prognostic value of this approach in primary mediastinal large B-cell lymphoma (PMBCL), a rare subtype of diffuse large B-cell lymphoma. They obtained histological verification in all of 17 patients with positive interim findings out of 36 consecutive patients with PMBCL having FDG PET examinations. Surprisingly enough, 16 of the 17 patients had negative histological results with inflammation and/or fibrosis. After a median follow-up of 48.5 months, 2 (12%) of 17 patients with positive- and 3 (16%) of 19 patients with negative interim PET findings developed disease progression. They concluded that a positive interim FDG PET result did not reflect active disease in the vast majority of PMBCL cases and that the relapse rate was similar regardless of the interim PET findings. They warned that interim FDG PET should be used with caution in PMBCL.

## Oncology—FMISO PET for hypoxia

Hypoxia is one of the most important factors for exacerbating malignancy, including gliomas and head and neck tumors. Hypoxia induces radioresistance and chemoresistance. Therefore, in vivo measurement of hypoxia in individual patients is of clinical interest. One of the most widely used PET radiotracers for hypoxia is [^18^F]fluoromisonidazole (FMISO), a nitroimidazole derivative.

Bekaert et al. [6] evaluated the relationship between uptake of FMISO within gliomas and hypoxia markers and with patient survival. In their study, FMISO uptake was associated with the expression of hypoxia markers such as hypoxia inducible factor-1, carbonic anhydrase 9, and vascular endothelial growth factor. Patients without FMISO uptake had a longer survival time than uptake positive patients. Toyonaga et al. [7] introduced new parameters, hypoxic metabolic tumor volume and total lesion glycolysis in hypoxia, using FMISO and FDG PET to determine the metabolically active hypoxic volume. These parameters are the volumes showing as FMISO-positive and FDG-positive. Their results showed that both hypoxic metabolic tumor volume and total lesion glycolysis in hypoxia were independent factors affecting survival in glioblastoma patients. Grkovski et al. [8] investigated the utility of dynamic FMISO PET for monitoring the early response to chemoradiotherapy in patients with head and neck cancer. They concluded that pharmacokinetic modeling of FMISO dynamic PET provided more detailed characterization of the tumor microenvironment and assessment of the response to chemoradiotherapy in head and neck cancer patients than did a single static image.

## Oncology—lymphoscintigraphy update

Lymphoscintigraphy is an indispensable examination which has to be done before performing sentinel lymph node biopsy (SLNB). SLNB has recently become a worldwide standard procedure for surgical treatment for various kinds of cancers, because it can abbreviate unnecessary regional lymph node dissection. SLNB seldom elicits a variety of complications such as lymphedema, motility disturbance etc., and it is an efficient method for the detection of metastatic lesions. Lymphoscintigraphy for detecting sentinel lymph nodes (SN) has progressed due to advances in nuclear imaging apparatus and techniques.

Borrelli et al. [9] evaluated whether the use of single-photon emission computed tomography/computed tomography (SPECT/CT) improved both visualization and anatomical localization of SN, and whether this additional information would have had any impact on the surgical approach and consequent retrieval of SN in patients with local breast cancer relapse (LBCR). They reported that the addition of SPECT/CT to the standard planar imaging protocol for lymphatic mapping and SN localization in LBCR patients appeared to improve detection of SN and their anatomical localization, and it led to better staging mainly in patients presenting drainage outside the ipsilateral axilla. Saad et al. [10] studied the role of SPECT/CT following 2-D planar lymphoscintigraphy in the detection and localization of SN in the groin. They reported that the addition of SPECT/CT provided superior images with increased nodal yield, more precise localization, a clearer distribution and drainage pattern, and a significant reduction in false extranodal hot spots observed on conventional planar imaging. Parredes et al. [11] evaluated the role and feasibility of indocyanine green (ICG)-^99m^Tc-nanocolloid tracer in SN detection in cervical cancer, and reported SLNB with ICG-^99m^Tc-nanocolloid to be feasible and safe in patients with early cervical cancer. The hybrid tracer provided bilateral SLN detection in all patients with a higher detection rate than blue dye, and so it could become an alternative to the current combined technique.

## Neurology—amyloid PET for Alzheimer’s disease

PET imaging ligands including [^11^C]Pittsburgh Compound B (PiB), [^18^F]florbetapir, [^18^F]flutemetamol, and [^18^F]florbetaben have been developed for estimation of cortical beta amyloid deposition in patients with Alzheimer’s disease. The ^18^F-labeled ligands have been approved as diagnostics in Europe, the United States and a few other countries. Although quantification of cortical uptake is helpful, it is treated as an adjunct to the visual interpretation in the official clinical setting. Recently, some articles have shown additional benefits of quantitative evaluation to improve the interpretation accuracy of amyloid PET images.

Yamane et al. [12] assessed the inter-rater variability of the visual interpretation of PiB PET images by three independent raters regarding the positivity/negativity of amyloid deposition that were obtained in a multicenter clinical research project, Japanese Alzheimer’s Disease Neuroimaging Initiative (J-ADNI). The results of visual interpretation were also compared with a semi-automatic quantitative analysis using mean cortical standardized uptake value ratio to the cerebellar cortex (mcSUVR). Inter-rater agreement was almost perfect in a total of 162 PiB PET scans, and positive or negative decision by visual interpretation was dichotomized by a cut-off value of mcSUVR = 1.5. As some cases of disagreement among raters tended to show low mcSUVR, referring to quantitative method may facilitate a correct diagnosis when evaluating images of low amyloid deposition. Pontecorvo et al. [13] examined the feasibility of using quantitation by three software packages to augment interpretation of florbetapir PET amyloid imaging. A total of 80 physician readers from three countries were trained on quantitation of florbetapir PET images and the principles for using quantitation to augment a visual read. Augmentation of visual interpretation of florbetapir PET amyloid images with quantitative information obtained using the commercially available software packages did not reduce the accuracy of readers who were already performing with above average accuracy on the visual read and may improve the accuracy and confidence of some readers in clinically relevant cases.

## Basic and translational molecular imaging

Many researchers have developed and utilized a variety of molecular imaging focusing on the urgent and continuing demand of novel types of PET probes for new biological targets to seek more specific and selective PET ligands for existing targets. These challenges and opportunities in PET molecular imaging provide more precise and rigorous methods for exploring disease models in greater depth.

Boron neutron capture therapy (BNCT) is one type of promising radiation therapy based on the nuclear reaction of ^10^B (*n*, α) ^7^Li with a higher cytotoxic effect by the emitted alpha particles. Watabe et al. [14] reported the usefulness of L-4-borono-2-[^18^F]fluoro-phenylalanine (FBPA) as a tumor-specific probe instead of L-paraboronophenylalanine (BPA) which is delivered to the cells through L-type amino acid transporter 1 (LAT1). They performed experiments using rat models of C6 glioma xenograft and of turpentine oil-induced subcutaneous inflammation. Studies revealed the higher selectivity of FBPA for LAT1. K1 and k2 values were significantly smaller in FBPA PET in inflammatory lesions. FBPA PET analysis based on the maximum standardized uptake value (SUVmax) showed high uptake in the tumor and relatively low uptake in inflammatory lesions.

*O*-(2-[^18^F]fluoroethyl)-L-tyrosine (FET) is an established amino acid tracer for brain tumor diagnosis in Western Europe. Stegmayr et al. [15] explored the relationship between FET uptake and blood–brain barrier (BBB) permeability. The microstructural changes of the BBB in tumor with and without glucocorticoid dexamethasone (Dex) treatment were also compared by transmission electron microscopy. In Dex treated rats Evans blue dye extravasation as the result of BBB disturbance was reduced significantly in 9L gliosarcoma and U87 glioblastoma xenograft models and slightly in F98 glioma xenograft models. Ultrastructural evaluation of tumor blood vessel endothelia revealed significant reduction of the cleft diameter between endothelial cells after Dex treatment in the F98 model. They concluded that FET uptake was not affected by BBB permeability or BBB restoration under supportive Dex therapy in brain tumor models, and that FET uptake in gliomas appeared to be widely independent of BBB permeability.

Human epidermal growth factor receptor type 2 (HER2)-directed therapy is useful and is the current standard of care for HER2-positive tumors such as breast and ovarian cancers. Pertuzumab is a HER2-targeting monoclonal antibody which has been approved by the Food and Drug Administration of the United States for patients with HER2-positive metastatic breast cancer. Non-invasive imaging of HER2 expression can allow physicians to monitor HER2-directed therapies in patients and can also be of assistance in patient stratification. Jiang et al. [16] showed the usefulness of ^64^Cu-labeled pertuzumab for in vivo imaging of HER2-positive ovarian cancer. In this study ^64^Cu-NOTA-pertuzumab showed high specificity in an ovarian cancer cell line with high HER2 expression. In subcutaneous tumors, PET imaging revealed tumor uptake to correlate well with HER2 expression levels.

## Conclusion

Written by associate editors of the Annals of Nuclear Medicine (ANM), this invited review article highlighted some of the high-quality research work that have been published in the European Journal of Nuclear Medicine and Molecular Imaging (EJNMMI) in 2017. Owing to Springer Nature, the publishing company of the both journals, subscribers of EJNMMI or ANM are allowed free access to the full text of all articles published in the two journals through our official websites. We hope that this review article will further influence our readers to access the full text of these highlighted articles and in this way arouse greater interest in recent European research trends in the field of nuclear medicine.
